# Boosting of Synaptic Potentials and Spine Ca Transients by the Peptide Toxin SNX-482 Requires Alpha-1E-Encoded Voltage-Gated Ca Channels

**DOI:** 10.1371/journal.pone.0020939

**Published:** 2011-06-09

**Authors:** Andrew J. Giessel, Bernardo L. Sabatini

**Affiliations:** Department of Neurobiology, Howard Hughes Medical Institute, Harvard Medical School, Boston, Massachusetts, United States of America; Institut National de la Santé et de la Recherche Médicale, France

## Abstract

The majority of glutamatergic synapses formed onto principal neurons of the mammalian central nervous system are associated with dendritic spines. Spines are tiny protuberances that house the proteins that mediate the response of the postsynaptic cell to the presynaptic release of glutamate. Postsynaptic signals are regulated by an ion channel signaling cascade that is active in individual dendritic spines and involves voltage-gated calcium (Ca) channels, small conductance (SK)-type Ca-activated potassium channels, and NMDA-type glutamate receptors. Pharmacological studies using the toxin SNX-482 indicated that the voltage-gated Ca channels that signal within spines to open SK channels belong to the class Ca_V_2.3, which is encoded by the Alpha-1E pore-forming subunit. In order to specifically test this conclusion, we examined the effects of SNX-482 on synaptic signals in acute hippocampal slices from knock-out mice lacking the Alpha-1E gene. We find that in these mice, application of SNX-482 has no effect on glutamate-uncaging evoked synaptic potentials and Ca influx, indicating that that SNX-482 indeed acts via the Alpha-1E-encoded Ca_V_2.3 channel.

## Introduction

Dendritic spines are small (∼1 micron diameter) morphological specializations stippled along the dendrites of principal neurons of the mammalian central nervous system [1]. In the hippocampus, each spine houses the post-synaptic density associated with a glutamatergic synapse and contains the protein machinery necessary to generate and regulate postsynaptic signals such as excitatory postsynaptic potentials (EPSPs) and spine head calcium (Ca) transients. Synaptic potentials and Ca transients are regulated by a non-linear signaling loop that is active in single dendritic spines and involves voltage-gated Ca channels (VGCCs), small conductance (SK)-type Ca-activated potassium channels, and NMDA-type glutamate receptors [2], [3], [4], [5], [6]. In brief, activation of AMPA- and NMDA-type glutamate receptors depolarizes the spine, which activates voltage-gated ion channels. Ca influx through VGCCs activates SK channels in the spine that, in turn, repolarize the spine and/or shunt depolarizing currents. Therefore, the net effect of SK channel opening is to decrease synaptic Ca influx and potentials. Conversely, reducing SK channel opening with the peptide toxin apamin or by activating muscarinic acetylcholine receptors enhances synaptic signals, thereby facilitating the induction of long-term potentiation [3], [4], [5], [6], [7], [8].

Previous work used pharmacological antagonists to block specific classes of VGCCs and identify the channel that provides the Ca that opens SK channels [3]. Application of SNX-482, a peptide toxin that blocks Ca_V_2.3 channels, was found to enhance synaptic potentials and Ca influx and prevent the effects of SK blockade. Thus, SNX-482 both mimicked and occluded the effects of SK channel blockade, indicating that SNX-482 sensitive VGCCs signal upstream of SK channels. In heterologous expression systems, SNX-482 specifically blocks Ca_V_2.3-type VGCCs encoded by the Alpha-1E gene, without effects on multiple other ion channels [9], [10]. For these reasons, the SNX-482-sensitive channel that regulates synaptic potentials and Ca transients in CA1 pyramidal neurons is likely to be Ca_V_2.3.

To test if the effects of SNX-482 on synaptic signaling are mediated by blockade of Ca_V_2.3 VGCCs, we examine the effects of SNX-482 in a knock-out mouse in which the Alpha-1E gene has been disrupted [11]. We find that, in contrast to our previous results from wild-type tissue, application of SNX-482 has no effect of glutamate-uncaging evoked synaptic potentials and Ca influx in acute hippocampal slices prepared from Alpha-1E knock-out animals.

## Results

**Figure 1:**
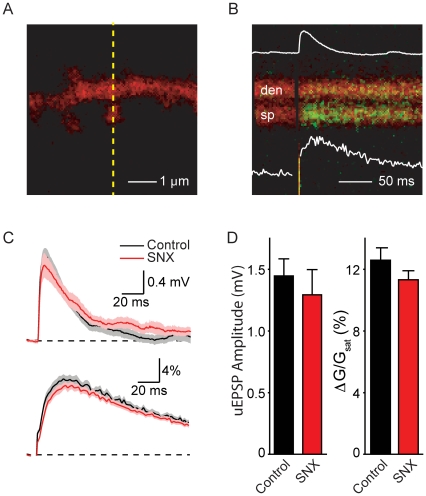
Uncaging-evoked synaptic responses in Alpha-1E knock-out mice are unaffected by SNX-482. (A) High-magnification image of a spiny dendrite of a CA1 hippocampal pyramidal cell formed from the red fluorescence of Alexa Fluor-594. The pyramidal neuron is in an acute slice cut from of an Alpha-1E knock-out mouse. (B) Example of fluorescence collected during a line scan, shown in the yellow line in (A), which intersects the dendrite (den) and spine head (sp) during glutamate uncaging at the spine head. The increase in green signal indicates a rise in intracellular [Ca]. The inset traces show the simultaneously recorded uEPSP (top, amplitude 1.2 mV) and the quantification of the green fluorescence in the spine head (bottom, amplitude 10% ΔG/G_sat_). (C) Average uEPSPs (top) and Ca-dependent changes in green fluorescence in the spine head (bottom) evoked by uncaging of glutamate in control conditions (black) and in the presence of SNX-482 (red). Data are shown as the mean (line) ± SEM (shaded region). (D) Summary of amplitudes of uEPSPs (left) and Δ[Ca]_spine_ (right) in control conditions (black) and in the presence of SNX-482 (red).

In order to determine if the effects of SNX-482 on uncaging-evoked potentials and Ca transients were mediated through Alpha-1E encoded VGCCs, we examined responses evoked by 2-photon laser uncaging of glutamate onto apical spines of CA1 pyramidal neurons from animals which were homozygous null for the Alpha1E subunit (Figure 1) [11]. Whole-cell current-clamp recordings were obtained from neurons in acute slices of the hippocampus (post-natal day 15–18) and 2-photon laser-scanning microscopy was used to image cellular and dendritic morphology as well as to monitor intracellular Ca transients (Figure 1A–B). Neurons were filled with a green-fluorescing Ca indicator (300 µM Fluo-5F) and a Ca-insensitive red fluorophore (10 µM Alexa Fluor-594) through a whole-cell recording electrode. Individual dendritic spines were stimulated by uncaging glutamate using 500 µs pulses of 725 nm laser light directed at a spot near the spine head (see methods). This resulted in an uncaging-evoked postsynaptic potential (uEPSP) detectable at the soma and increases in green fluorescence (ΔG/G_sat_, see methods) in the spine head, indicative of elevated [Ca] (Δ[Ca]_spine_, Figure 1B). Analysis was limited to spines with clearly defined heads that were well separated from the parent dendrite and located less than 150 µm from the soma on radial oblique dendrites in order to use identical experimental conditions as in previous studies [2], [3], [5].

### Uncaging responses in Alpha 1E KO mice are unaffected by SNX-482

Uncaging-evoked potentials and Ca transients were measured in control conditions and in the presence of the Ca channel antagonist SNX-482 (0.3 µM) (Figure 1C). In the presence of SNX-482, neither the uEPSP nor Δ[Ca]_spine_ amplitudes were significantly different from control (control: n = 20; uEPSP: 1.47±0.14 mV; spine ΔG/G_sat_: 12.6±0.8%; SNX-482: n = 20; uEPSP: 1.3±0.2 mV; spine ΔG/G_sat_: 11.4±0.58%, Figure 1D). Uncaging laser power used to stimulate control spines was not significantly different than that used in the presence of SNX-482 as measured by photobleaching of Alexa 594 in the spine head (control: 44.8±3%; SNX-482: 37.4±2.6% bleaching). These results are in contrast to our those from previous studies in wild-type mice in which the presence of SNX-482 boosted the amplitudes of the uEPSP and Δ[Ca]_spine_ approximately 2-fold relative to in control conditions.

## Discussion

We have shown that SNX-482 has no effect on uncaging-evoked potentials and Δ[Ca]_spine_ in hippocampal CA1 pyramidal neurons of mice lacking the Alpha-1E-encoded VGCC. This suggests that the primary target of this toxin that regulates postsynaptic signaling at the CA3 to CA1 synapse is the Ca_V_2.3 VGCC. These results are in contrast to those of previous studies in hippocampal slices of wild-type mice in which application of SNX-482 increased the amplitudes of uEPSPs and the Δ[Ca]_spine_ [3], [5]. Thus, the present data are consistent with the interpretation that the effects of SNX-482 in wild-type mice are due to blockade of Alpha-1E encoded Ca_V_2.3 VGCC [2], [3], [5].

Although direct comparison of parameters across mouse genotypes may be difficult, it is noteworthy that the uEPSP amplitudes in control conditions in Alpha-1E knock-out tissue measured in this study are ∼2-fold larger than control uEPSPs in C57/BL6 mice (∼0.85 mV), and similar to the size of uEPSPs in C57/BL6 mice in the presence of SNX-482 or apamin (∼1.5 mV) [2], [3], [5], [6]. This basal increase may reflect enhanced synaptic signaling due to the uncompensated loss of Ca_V_2.3 channels and failure to activate SK channels during synaptic potentials. Nevertheless, future studies are necessary to directly address if the enhancement of synaptic signals via blockade of SK channels is prevented by deletion of the Alpha-1E gene.

## Materials and Methods

### Animal Handling and Slice Preparation

Animals were handled according to protocols that were approved by the Harvard Standing Committee on Animal Care (#03551) and that are in accordance with Federal guidelines. The specific approved protocol for this study was euthanasia under anesthesia followed by tissue harvest. Post-natal day 15–18 Alpha1E KO mice [11] were anesthetized by inhalation of isoflurane and euthanized by decapitation. Transverse hippocampal slices were prepared as described previously [3] in a cold choline-based artificial cerebrospinal fluid (choline-ACSF) containing (in mM): 25 NaHCO_3_, 1.25 NaH_2_PO_4_, 2.5 KCl, 7 MgCl_2_, 25 glucose, 1 CaCl_2_, 110 choline chloride, 11.60 ascorbic acid, and 3.10 pyruvic acid, and equilibrated with 95% O_2_/5% CO_2_. Slices of 300 µm thickness were cut with a Leica VT1000s (Leica Instruments, Nussloch, Germany) and transferred to a holding chamber containing ACSF consisting of (in mM): 127 NaCl, 2.5 KCl, 25 NaHCO_3_, 1.25 NaH_2_PO_4_, 2.0 CaCl_2_, 1.0 MgCl_2_, and 25 glucose, equilibrated with 95% O_2_/5% CO_2_. Slices were incubated at 32°C for 30–45 min and then left at room temperature (20–22°C) until recordings were performed. All recordings were performed within 4 hours of slice cutting in a submerged slice chamber perfused with ACSF warmed to 32°C and equilibrated with 95% O_2_/5% CO_2_.

### Electrophysiology

Whole-cell recordings were made from CA1 pyramidal neurons visualized under infrared differential interference contrast (IR-DIC) on a combined 2-photon imaging and uncaging microscope [12]. Patch pipettes (open pipette resistance 2.5–4.5 MΩ) were filled with an internal solution containing (in mM): 135 KMeSO_4_, 10 HEPES, 4 MgCl_2_, 4 NaATP, and 0.4 Na_2_GTP, and 10 Na_2_Creatine Phosphate (pH 7.3). 300 µM Fluo-5F (Molecular Probes, K_D_∼1.1 µM) and 10 µM Alexa 594 (Molecular Probes) were included in the internal solution.

Recordings were made with an Axoclamp 200B amplifier (Axon Instruments, Union City, CA). Data were filtered at 5 kHz and sampled at 10 kHZ. Current was injected to hold cells at approximately −70 mV in current-clamp mode. Cells were rejected if holding currents exceed −100 pA. Series (not compensated) and input resistances were measured throughout the experiment, and recordings were discarded if series resistance exceeded 20 MΩ. D-Serine (10 µM) was included in the ACSF to reduce NMDAR desensitization that can occur during glutamate uncaging. Liquid junction potentials of ∼8 mV were not corrected.

### Pharmacology

When appropriate and as listed in the text, pharmacological agents were used in the extracellular solution at the following final concentrations (in µM): 10 D-serine (Sigma-Aldrich, St. Louis, MO), 0.3 SNX-482 (Peptides International).

### 2-Photon imaging and uncaging

The experiments were performed using a custom built 2-photon laser scanning microscope based on a BX51Wl microscope (Olympus) as described previously [12]. Two Ti-sapphire lasers (Mira/Verdi, Coherent) tuned to 840 and 725 nm were used for imaging and glutamate uncaging, respectively. In all uncaging experiments 3.75 mM MNI-glutamate (Tocris Cookson, Ellisville, MO) was included in a small volume (∼9 ml) of re-circulating ASCF. Uncaging laser pulse duration was 0.5 ms and power delivered to each spine was set to bleach ∼40% of the red fluorescence in the spine head as described previously [2], [3], [5]. After laser power was set, the periphery of each spine was probed to identify the uncaging spot that triggered the largest somatic response and uncaging at this spot was used for the remainder of the analysis [13]. Image and electrophysiology acquisition was controlled by custom software written in MATLAB (Mathworks) and data analysis was performed using custom software written in Igor Pro (Wavemetrics).

Cells were filled with two fluorescent dyes: a Ca-insensitive fluorophore (Alexa Fluor 594), which fluoresces in the red, and a Ca-sensitive fluorophore (Fluo-5F), which fluoresces in the green. Red fluorescence was used to identify spines and dendrites. Relative differences in the amplitude of intracellular Ca transients were judged by comparing the amplitude of green fluorescence transients as described previously [2], [3], [5].

### Data Analysis and Statistics

The amplitudes of uncaging-evoked potentials were measured as averages around their peak values. The peaks of uncaging-evoked Ca transients were calculated by averaging 20–50 ms after the uncaging pulse. All data are expressed as the mean ± SEM. Average traces are shown as the mean (line) ± the SEM (shaded regions or bars). A two-tailed t-test was used to determine significance of differences in uEPSPs and ΔG/G_sat_ across conditions. p<0.05 was considered significant.
